# How to Live

**Published:** 1879-09

**Authors:** 


### HOW TO LIVE.

A wealthy gentleman of Boston, several years ago, gave the editor of the *Worcester Palladium* a short narration of his own experience. He had an income of $10,000 a year, (a large sum then, but not considered so now,) a house in town, and country-seat a few miles out. He had several children—a coach, fine horses and a driver; and took pleasure in riding every day with his children.

One day, when riding, the thought struck him that each one of his children would expect to have a fine house, and coach, and horses and driver, as their father had before them, and to live as he lived; and if they did not, they would be unhappy. He did not think that all of them could have things as he had them, or live as he was living; and he rode home; sent his coach and horse to market, and sold them; bought a cheap carry-all, and became his own driver.

With emphasis he declared that no amount of wealth could induce him to return to his former mode of living, for if any of his children should chance to be poor, as in all probability some of them would be, they should not suffer in their feeling by the reflection that their father rode in his coach while they had to rough it on foot. The example he gave them afforded him satisfaction greater than his wealth had to bestow.

Parents oppose liberal salaries for good teachers and for supplying teachers with useful apparatus. They do not manifest as much interest in caring for the health and improvement of their children while in school as in looking after their live stock. They should visit the schools, assist the teachers in understanding the peculiarities of the children, should help the teachers, instead of finding fault, which is generally the only recognition and attention the school receives.

